# Salt Potentiates Methylamine Counteraction System to Offset the Deleterious Effects of Urea on Protein Stability and Function

**DOI:** 10.1371/journal.pone.0119597

**Published:** 2015-03-20

**Authors:** Safikur Rahman, Md. Tabish Rehman, Laishram R. Singh, Marina Warepam, Faizan Ahmad, Tanveer Ali Dar

**Affiliations:** 1 Centre for Interdisciplinary Research in Basic Sciences, Jamia Millia Islamia, New Delhi, India; 2 Dr. B. R. Ambedkar Center for Biomedical Research, University of Delhi, Delhi, India; 3 Clinical Biochemistry, University of Kashmir, Srinagar, Jammu & Kashmir, India; Emory University, UNITED STATES

## Abstract

Cellular methylamines are osmolytes (low molecular weight organic compounds) believed to offset the urea’s harmful effects on the stability and function of proteins in mammalian kidney and marine invertebrates. Although urea and methylamines are found at 2:1 molar ratio in tissues, their opposing effects on protein structure and function have been questioned on several grounds including failure to counteraction or partial counteraction. Here we investigated the possible involvement of cellular salt, NaCl, in urea-methylamine counteraction on protein stability and function. We found that NaCl mediates methylamine counteracting system from no or partial counteraction to complete counteraction of urea’s effect on protein stability and function. These conclusions were drawn from the systematic thermodynamic stability and functional activity measurements of lysozyme and RNase-A. Our results revealed that salts might be involved in protein interaction with charged osmolytes and hence in the urea-methylamine counteraction.

## Introduction

The concentration of urea in the intracellular and extracellular environment (including human kidney cells) is high enough to destabilize or inhibit many proteins, yet organisms survive and function [1,2]. Urea is a waste product of cellular metabolic activities and used as an osmolyte (low molecular weight compound to protect macromolecules from stress). Concentrations of urea in mammalian kidney are as high as 400–600 mM, which under antidiruetic condition could elevate up to 3–4 M [2]. Urea is a common denaturant; it alters melting temperature (*T*_m_) of proteins [3,4] by disrupting non-covalent interactions responsible for the globular protein structure [3,5,6,7], and has hostile effects on enzyme activity parameters (*V*_max_ and *K*_m_) [5,6]. To remove the myriad effects of urea on macromolecular stability and function, organisms evolved to have a unique class of osmolyte system, especially methylamine compounds [8,9,10]. Trimethylamine N-oxide (TMAO), glycine betaine (betaine) and sarcosine are the major methylamine osmolytes used by organisms [8,9] in urea-rich cells of various organisms. Cellular concentrations of the two osmolytes are roughly 2:1 molar ratio in tissues of urea-rich cells [6,8,11]. It is believed that at this ratio there is complete algebraic additivity between the urea and methylamine in terms of thermodynamic and functional activity of proteins [8].

The mechanism of urea methylamine counteraction on protein stability and function has been well investigated. Transfer free energy measurements of the protein groups to urea-methylamine solutions [12], preferential interaction measurements of proteins in the presence of these osmolytes [13], and enzyme activity measurements in the presence of urea and methylamines have uncovered several insights of urea-methylamine interactions with proteins. It is known that urea destabilizes proteins by its favorable interactions (due to preferential binding) with the peptide backbone while addition of methylamine opposes the effect (by enhancing preferential hydration effect) caused by urea [12,14,15,16]. Despite these progresses, the urea methylamine counteraction system has failed or worked partially on significant number of enzymes tested so far ([17] and references therein) [4,18]. To date the raison d’eter for the partial counteraction and failure of counteraction has been an unattended avenue. In fact, failure or partial counteraction in terms of thermodynamic stability and functional activity will pose serious threats for survival of organisms if the cells contain high amount of urea.

There is no consensus on the types of solutes and the osmotic balance between the intracellular and extracellular environments in the mammalian kidney cells due to urine concentrating mechanism and in marine invertebrates due to high extracellular salt. However, in mammalian kidney, medullar fluid osmolarity increases up to 3800 mosmol l^-1^ (equivalent to ~2.0 M NaCl) during antidiuresis and decreases up to 170 mosmol l^-1^ during diuresis [19]. Interestingly, several reports indicate that cellular salt, NaCl/KCl is co-accumulated at very high concentrations with urea [19,20,21,22,23,24,25]. Furthermore, a good correlation is observed between the rise and fall of urea and the amount of salt in many tissues [26]. In the light of all these observations, we thought that salt (NaCl) might be another osmotic agent that potentiates methylamine osmolyte system for perfect counteraction of urea’s effect on protein stability and function. We discovered that complete thermodynamic counteraction between urea and methylamines at 2:1 ratio on protein stability and function happens only in the presence of NaCl. The present study provides an evidence for the possible involvement of cellular salts in protein-osmolyte interactions.

## Materials and Methods

Lyophilized preparations of RNase-A (type III-A) and hen egg white lysozyme were purchased from Sigma Chemical Company (St Louis, MO, USA). Cytidine 2`-3`cyclic monophosphate (C > P), *M*. *luteus* cells, sarcosine and TMAO were also procured from the same. Urea and NaCl were ultra pure samples from Schwarz/Mann. These and other chemicals, which were of analytical grade, were used without further purification.

### Protein solution preparation

Lysozyme and RNase-A solutions were dialyzed extensively against 0.1 M KCl at pH 7.0 and 4°C. Protein stock solutions were filtered using 0.22-μm Millipore filter syringe. Both proteins gave single bands during polyacrylamide gel electrophoresis (data not shown). Concentration of proteins was determined experimentally using molar absorption coefficient (M^–1^ cm^–1^) values of 39000 at 280 nm for lysozyme [27] and 9800 at 277.5 nm for RNase-A [28]. Degassed buffer containing 0.1M KCl was used to prepare all our desired solutions. The pH of the protein solutions changes upon the addition of urea, urea-NaCl or urea-methylamine-NaCl mixtures; to adjust the required pH HCl or NaOH was added to the solution. Since the change in pH may also occur upon heating, the pH of the solution was, therefore, measured after the denaturation experiment. It has been observed that the change in pH was not significant.

### Thermal denaturation

Thermal denaturation studies were carried out in a Jasco V-660 UV/visible spectrophotometer equipped with a Peltier-type temperature controller (ETCS-761) with a heating rate of 1°C/min. This scan rate was found to provide adequate time for equilibration. Each sample was heated from 20 to 85°C. The change in absorbance with increasing temperature was followed at 300 nm for lysozyme and 287 nm for RNase-A.

About 650 data points of each transition curve were collected. After denaturation, the sample was immediately cooled down to measure reversibility of the reaction. All blanks solution showed negligible change in absorbance with temperature and were, therefore, neglected during the data analysis. The raw absorbance data were converted into a molar absorption coefficient (M^–1^ cm^–1^) at a given wavelength. Each heat-induced transition curve was analyzed for *T*_m_ (midpoint of denaturation) and Δ*H*_m_ (enthalpy change at *T*_m_) using a non-linear least squares method according to the relation,

$$y\text{(}T\text{)}=\frac{y_{\text{N}}(T)+y_{\text{D}}\text{(}T\text{) exp [-}\Delta H_{\text{m}}/R\text{(1/}T\text{- 1/}T_{\text{m}}\text{)]}}{\text{1 }+\text{ exp[-}\Delta H_{\text{m}}\text{/}R\text{(1/}T\text{ - 1/}T_{\text{m}}\text{)]}}$$ (1)

where *y*(*T*) is the optical property at temperature *T* (Kelvin), *y*_N_(*T*) and *y*_D_(*T*) are the optical properties of the native and denatured protein molecules at *T* K, respectively, and *R* is the gas constant. In the analysis of the transition curve, it was assumed that a parabolic function describes the dependence of the optical properties of the native and denatured protein molecules i.e., *y*_N_(*T*) = *a*_N_ + *b*_N_ *T* + *c*_N_ *T* ^2^, and *y*_D_(*T*) = *a*_D_ + *b*_D_ *T* + *c*_D_ *T* ^2^, where *a*_N_, *b*_N_, *c*_N_, *a*_D_, *b*_D_, and *c*_D_ are temperature-independent coefficients [29]. A plot of *H*_m_ versus *T*_m_ at each co-solvent concentration gave the value of *C*_p_, the constant-pressure heat capacity change (*C*_p_ = (*H*_m_/*T*_m_)_p_). Δ*G*_D_ (T), the value of Δ*G*_D_ at any temperature *T*, was estimated with the help of the Gibbs-Helmholtz equation (Equation 2) with observed values of *T*_m_, *H*_m_, and *C*_p_.

$$\Delta G_{\text{D }}(T)=\Delta H_{\text{m}}\left(\frac{T_{\text{m}}\text{–}T}{T_{\text{m}}}\right)-\Delta C_{\text{p}}\left[(T_{\text{m}}-T)+T\ln\left(\frac{T}{T_{\text{m}}}\right)\right]$$ (2)

### Functional activity measurements

#### Lysozyme activity

The effect of urea, TMAO, sarcosine, urea-methylamines mixtures and urea-methylamine-NaCl mixtures on the kinetic parameters (*K*_m_ and *k*_cat_) of the lytic activity of lysozyme towards *M*. *luteus* cell walls was assayed at pH 7.0 and 25°C by the method of Maurel & Douzou, 1976 [30]. The substrate and the enzyme were pre-incubated in a given concentration of each osmolyte, alone or in combination. The decrease in optical density of a turbid aqueous suspension of *M*. *luteus*, following the addition of lysozyme, was recorded at 450 nm in a Jasco V-660 UV/Visible spectrophotometer with constant stirring. The rate of lysis was deduced from the slope of the linear part of the recordings, usually over the first 30 sec, wherein 10 to 20% of the substrate was lysed. The molar absorption of an aqueous suspension of *M*. *luteus* cell walls was ε_405_ = 0.65 X 10^-2^ mg l^-1^at 450 nm. The rate of lysis was defined as the weight of cells lysed (in mg ml^-1^) per sec and per mol of lysozyme. The assay media were directly prepared in a 1 cm path length glass cell. In a typical experiment, 10 to 200 μl, depending on the desired concentration, of a stock aqueous suspension of *M*. *luteus* cells (3 mg m1^-1^) were added and the final volume was adjusted to 3 ml with 50 mM cacodylate buffer, pH 7.0. The cells were then placed into the spectrophotometer cell holder set at 25 ± 0.1°C. The reaction was initiated by the addition with stirring of 20 μl of a lysozyme stock solution (1 mg/ml, in 50 mM cacodylate buffer, pH 7.0). The kinetic parameters *K*_m_ (μg m1^-1^) and *k*_cat_ (mg ml^-1^ s^-1^ M^-1^) were determined from Michaelis-Menten plots using Equation 3. In these experiments *M*. *luteus* concentration was varied from 10 to 200 μg m1^-1^ and the lysozyme concentration was kept constant at 0.45 μM.

#### RNase-A activity

RNase-A activity was assayed to see the effect of urea, TMAO, sarcosine, urea-methylamine mixtures and urea-methylamine-NaCl mixtures on its kinetic parameters (*K*_m_ and *k*_cat_) as described previously [31]. For this, the substrate (cytidine 2`-3`cyclic monophosphate) and enzyme were pre-incubated in a given concentration of each osmolyte, alone or in combination. RNase-A mediated hydrolysis of the substrate in concentration range 0.05–0.50 mg ml^-1^ was followed by measuring the change in absorbance at 292 nm at 25.0 ± 0.1°C for 20 min in Jasco V-660 UV⁄ Vis spectrophotometer. From each progress curve at a given substrate concentration, initial velocity (v) was determined from the linear portion of the progress curve, usually first 30 sec. The plot of initial velocity (v) versus [S] (in mM) at each osmolyte concentration was analyzed for *K*_m_ and *k*_cat_ using Equation (3).

$$v=\frac{{\text{V}}_{\text{max}}\left[\text{S}\right]}{K_{\text{m}}+\left[\text{S}\right]}$$ (3)

Where *ν* is the initial velocity and [S] is the substrate concentration.

## Results

**Fig 1:**
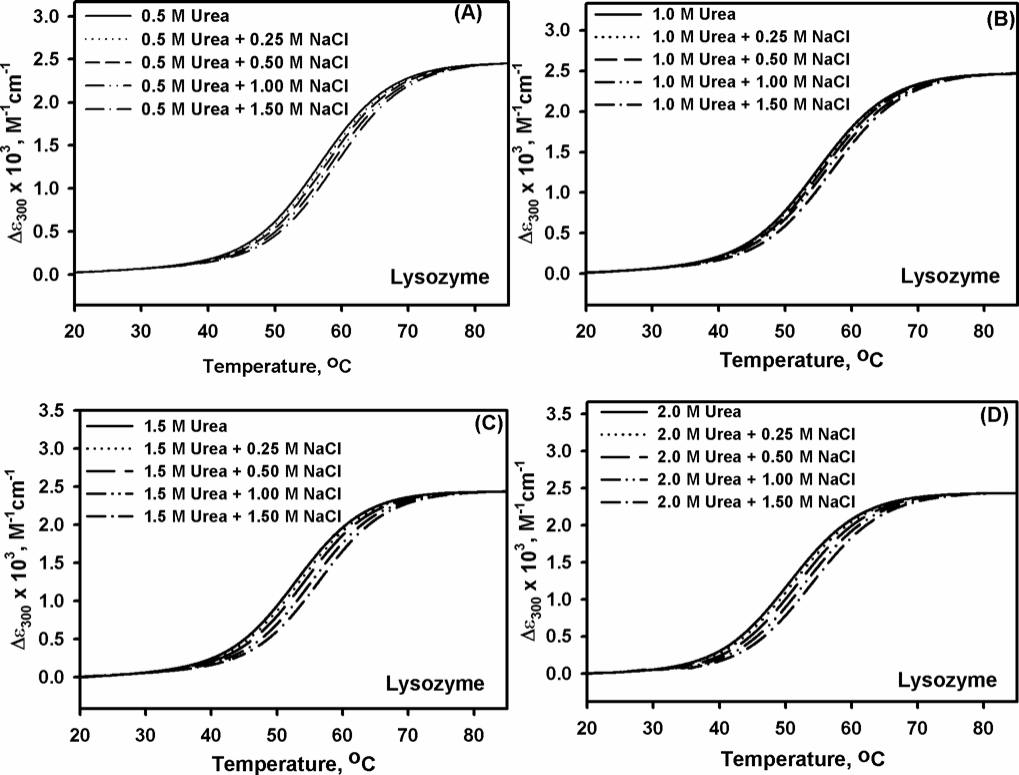
Combined effect of urea and salt on lysozyme. Representative thermal denaturation curves of lysozyme at pH 7.0 in the presence of different concentrations of urea-NaCl mixtures. Panels (A)–(D) show transition curves obtained in the presence of 0.50, 1.00, 1.50 and 2.00 M urea respectively with the indicated concentrations of NaCl.

**Fig 2:**
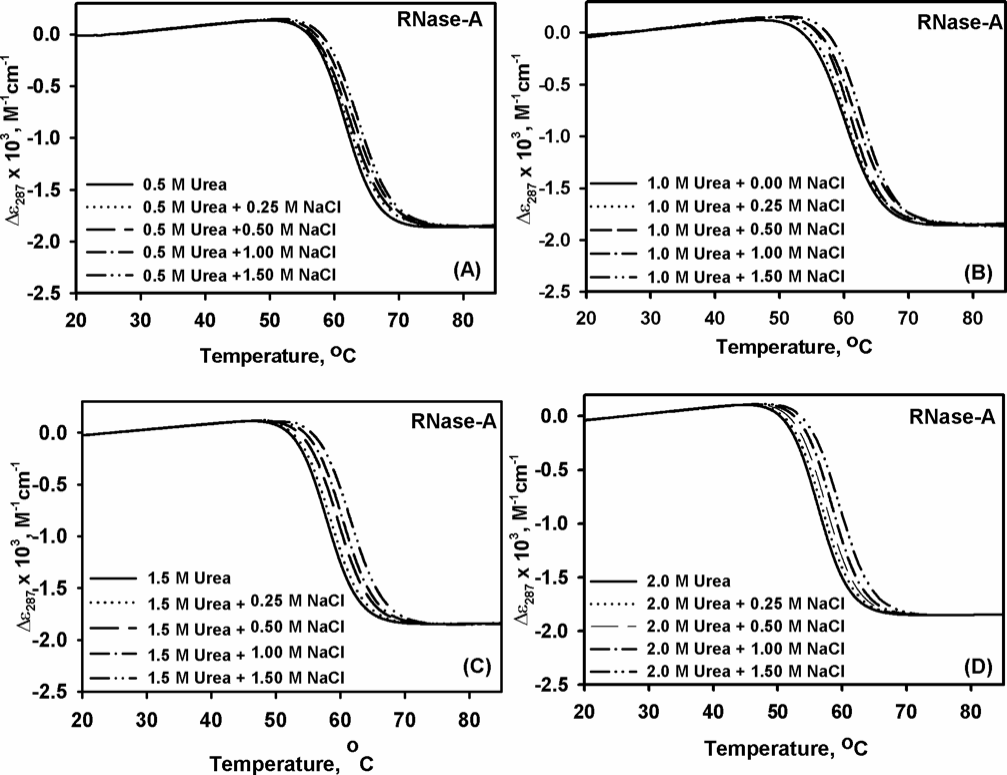
Combined effect of urea and salt on RNase-A. Representative thermal denaturation curves of RNase-A at pH 7.0 in the presence of different concentrations of urea-NaCl mixtures. Panels (A)–(D) show transition curves obtained in the presence of 0.50, 1.00, 1.50 and 2.00 M urea respectively with the indicated concentrations of NaCl.

In our previous study, we observed that methylamines (TMAO and Sarcosine) exhibit partial counteraction of the urea’s effect on the lysozyme stability [4]. In the present study, we have further investigated the effect of urea-methylamine counteraction behavior on the stability of RNase-A (Table 1) by following the same procedure described earlier [4]. Similar to lysozyme we observed partial nature of counteraction behavior. We have further investigated the effect of NaCl on urea-induced protein destabilization by measuring heat-induced denaturation of lysozyme and RNase-A (at pH 7.0) in the presence of different concentrations of NaCl. For this, we destabilize the proteins (RNase-A and lysozyme) with different concentrations of urea (0.5, 1.0, 1.5, 2.0 M) and titrated each of them with different concentrations of NaCl (0.25, 0.50, 1.0, 1.5 M). We have intentionally chosen these urea concentrations since both enzymes exist in native state in the presence of these concentrations. The transition curves were measured at least in triplicate, and found reversible. Figs. 1 and 2 show the representative heat-induced denaturation curves of lysozyme and RNase-A respectively in the presence of different urea-NaCl mixtures. Each heat-induced denaturation curve was analyzed for Δ*H*_m_ and *T*_m_ according to equation 1 with all eight free parameters (*a*_N_, *b*_N_, *c*_N_, *a*_D_, *b*_D_, *c*_D,_ *T*_m_ and Δ*H*_m_). The thermodynamic parameters evaluated in this manner are given in Table 2. S1 Table also shows the respective control of thermodynamic parameters obtained in the presence of NaCl alone (i.e. in the absence of urea). It is seen in S1 Table and S2 Table that NaCl stabilizes the protein in the absence and presence of urea. It should, however, be noted that thermal denaturation of lysozyme was always measured in the presence of 2.0 M GdmCl as complete transition could not be obtained in the measurable temperature range. Values of Δ*H*_m_ and *T*_m_ of lysozyme were corrected for the contribution due to the presence of 2.0 M GdmCl following the procedure describe earlier [32]. This correction involves (i) measurements of heat-induced denaturation curves in the presence of 1.5, 2.0, 2.5, 3.0 and 3.5 M GdmCl (results not shown); (ii) analysis of each denaturation curve for Δ*H*_m_ and *T*_m_ using equation 1; (iii) construction of plots of Δ*H*_m_ versus [GdmCl] and *T*_m_ versus [GdmCl] (plots not shown); (iv) determination of *T*_m_ and Δ*H*_m_ at 0 M [GdmCl]; and (v) estimation of correction factor for Δ*H*_m_ and *T*_m_ which are equal to Δ*H*_m_ (0 M GdmCl)- Δ*H*_m_(2 M GdmCl) and *T*_m_(0 M GdmCl)-*T*_m_ (2 M GdmCl) respectively. Hence, the *T*_m_ and Δ*H*_m_ presented in Table 1 for lysozyme is the corrected values for the contribution of GdmCl.

**Table 1:** Thermodynamic parameters associated with the thermal unfolding of lysozyme and RNase-A under different conditions at pH 7.0.

	Lysozyme^a^			RNase-A^b^		
Conc. (M)	Δ*G*_D_° (kcal/mol)	*T*_m_ (°C)	Δ*H*_m_ (kcal/mol)	Δ*G*_D_° (kcal/mol)	*T*_m_ (°C)	Δ*H*_m_ (kcal/mol)
0.00	13.20 ± 0.31	86.0 ± 0.2	130 ± 3	10.21 ± 0.13	63.6 ± 0.3	114 ± 2
			**Urea**			
0.50	12.57 ± 0.26	83.0 ± 0.2	127 ± 4	9.01 ± 0.15	61.6 ± 0.2	106 ± 3
1.00	11.80 ± 0.25	79.5 ± 0.3	123 ± 2	8.53 ± 0.21	59.8 ± 0.3	104 ± 3
1.50	11.22 ± 0.22	76.8 ± 0.1	120 ± 3	7.81 ± 0.21	57.7 ± 0.4	100 ± 4
2.00	10.75 ± 0.21	73.8 ± 0.2	118 ± 3	7.40 ± 0.15	56.3 ± 0.2	98 ± 2
			**TMAO**			
0.25	13.88 ± 0.29	87.1 ± 0.2	134 ± 2	10.61 ± 0.11	64.1 ± 0.2	115 ± 3
0.50	14.44 ± 0.34	88.3 ± 0.1	137 ± 3	10.98 ± 0.21	64.9 ± 0.3	117 ± 2
0.75	15.20 ± 0.27	90.0 ± 0.3	141 ± 4	11.35 ± 0.18	65.7 ± 0.2	119 ± 3
1.00	15.57 ± 0.35	91.9 ± 0.2	145 ± 2	11.86 ± 0.15	66.8 ± 0.4	122 ± 4
			**Sarcosine**			
0.25	13.72 ± 0.35	97.6 ± 0.1	133 ± 2	10.63 ± 0.13	64.8 ± 0.2	116 ± 2
0.50	14.44 ± 0.25	88.3 ± 0.3	137 ± 4	10.93 ± 0.10	65.2 ± 0.3	118 ± 3
0.75	15.02 ± 0.34	89.3 ± 0.2	140 ± 3	11.22 ± 0.14	66.3 ± 0.3	119 ± 3
1.00	15.55 ± 0.28	90.0 ± 0.3	143 ± 4	11.61 ± 0.19	67.2 ± 0.2	121 ± 2
			**Urea + TMAO**			
0.50 + 0.25	12.94 ± 0.25	84.0 ± 0.2	129 ± 2	9.80 ± 0.11	63.0 ± 0.2	111 ± 2
1.00 + 0.50	12.72 ± 0.24	82.5 ± 0.3	128 ± 4	9.22 ± 0.12	62.3 ± 0.3	107 ± 3
1.50 + 0.75	12.01 ± 0.19	81.0 ± 0.1	125 ± 3	8.77 ± 0.14	61.5 ± 0.3	104 ± 3
2.00 + 1.00	11.62 ± 0.22	79.0 ± 0.3	122 ± 2	8.53 ± 0.19	60.6 ± 0.3	102 ± 3
			**Urea + Sarcosine**			
0.50 + 0.25	12.82 ± 0.27	85.8 ± 0.2	128 ± 3	9.78 ± 0.12	62.3 ± 0.3	112 ± 3
1.00 + 0.50	12.77 ± 0.17	83.7 ± 0.3	125 ± 2	9.30 ± 0.14	61.8 ± 0.2	108 ± 3
1.50 + 0.75	11.91 ± 0.29	82.5 ± 0.2	123 ± 4	9.01 ± 0.11	61.2 ± 0.2	106 ± 4
2.00 + 1.00	11.55 ± 0.28	81.0 ± 0.4	121 ± 2	8.76 ± 0.13	60.5 ± 0.3	104 ± 2

^a^ Values taken from [4].

^b^Values were obtained from the measurements and analysis of thermal denaturation curves using the same procedures described in [4].

**Table 2:** Thermodynamic parameters associated with the thermal unfolding of lysozyme and RNase-A in the presence of different concentrations of urea- NaCl mixture at pH 7.0.

Lysozyme				RNase-A		
Urea + NaCl (M)	Δ*G*_D_° (kcal/mol)	*T*_m_ (°C)	Δ*H*_m_ (kcal/mol)	Δ*G*_D_° (kcal/mol)	*T*_m_ (°C)	Δ*H*_m_ (kcal/mol)
0.00 + 0.00	13.19 ± 0.31	86.0 ± 0.2	130 ± 3	10.21 ± 0.13	63.6 ± 0.3	114 ± 2
0.50 + 0.00	12.56 ± 0.26	83.0 ± 0.2	127 ± 4	9.01 x 0.19	61.6 ± 0.2	106 ± 4
0.50 + 0.25	12.56 ± 0.32	84.3 ± 0.4	127 ± 2	9.10 ± 0.17	61.9 ± 0.4	106 ± 3
0.50 + 0.50	12.81 ± 0.27	84.8 ± 0.2	129 ± 2	9.30 ± 0.14	62.5 ± 0.3	108 ± 3
0.50 + 1.00	13.26 ± 0.27	85.5 ± 0.3	131 ± 3	9.76 ± 0.12	63.0 ± 0.3	111 ± 4
0.50 + 1.50	13.68 ± 0.28	86.0 ± 0.2	133 ± 2	10.10 ± 0.14	63.5 ± 0.2	113 ± 2
1.00 + 0.00	11.90 ± 0.25	79.5 ± 0.3	123 ± 2	8.53 ± 0.14	59.8 ± 0.3	104 ± 4
1.00 + 0.25	11.91 ± 0.28	80.0 ± 0.2	124 ± 2	8.52 ± 0.15	60.1 ± 0.2	104 ± 4
1.00 + 0.50	12.10 ± 0.32	80.6 ± 0.3	125 ± 3	8.72 ± 0.19	61.0 ± 0.2	105 ± 2
1.00 + 1.00	12.65 ± 0.29	81.2 ± 0.2	127 ± 3	9.22 ± 0.14	61.6 ± 0.4	108 ± 3
1.00 + 1.50	13.12 ± 0.24	84.6 ± 0.3	129 ± 4	9.73 ± 0.17	62.9 ± 0.3	110 ± 4
1.50 + 0.00	11.22 ± 0.22	76.8 ± 0.1	120 ± 3	7.81 ± 0.21	57.7 ± 0.4	100 ± 4
1.50 + 0.25	11.49 ± 0.32	80.7 ± 0.4	120 ± 2	7.99 ± 0.17	58.3 ± 0.4	101 ± 3
1.50 + 0.50	11.73 ± 0.27	81.4 ± 0.2	123 ± 2	8.14 ± 0.14	59.4 ± 0.3	102 ± 3
1.50 + 1.00	12.35 ± 0.27	82.5 ± 0.3	126 ± 3	8.75 ± 0.12	60.2 ± 0.3	105 ± 4
1.50 + 1.50	12.81 ± 0.28	83.6 ± 0.2	129 ± 2	9.29 ± 0.14	61.4 ± 0.2	118 ± 2
2.00 + 0.00	10.75 ± 0.21	73.8 ± 0.2	118 ± 3	7.40 ± 0.15	56.3 ± 0.2	98 ± 2
2.00 + 0.25	11.11 ± 0.25	78.8 ± 0.2	119 ± 2	7.50 ± 0.15	56.9 ± 0.2	100 ± 4
2.00 + 0.50	11.35 ± 0.30	79.6 ± 0.3	121 ± 3	7.79 ± 0.19	57.6 ± 0.2	101 ± 2
2.00 + 1.00	11.96 ± 0.29	80.7 ± 0.2	124 ± 3	8.43 ± 0.14	58.5 ± 0.4	104 ± 3
2.00 + 1.50	12.52 ± 0.21	81.9 ± 0.3	127 ± 4	8.92 ± 0.17	59.7 ± 0.3	106 ± 4

All the 12 values of *T*_m_ and Δ*H*_m_ obtained from three independent measurements of four different [urea] were used to make a plot of Δ*H*_m_ versus *T*_m_. Such plots were analyzed for Δ*C*_p_(Δ*C*_p_ = (δΔ*H*_m_/δ*T*_m_)_p_). Δ*C*_p_ values evaluated in this manner for lysozyme and RNase-A in the absence of NaCl and urea were 1.63 ± 0.08 and 1.24 ± 0.04 kcal mol^-1^K^-1^ respectively. These values are in good agreement with the earlier reports on these proteins [4]. No significant change in Δ*C*_p_ of proteins was observed in the presence of NaCl-urea mixtures in the concentration ranges used in this study. We have thus used Δ*C*_p_ values of 1.63 ± 0.08 and 1.24 ± 0.04 kcal mol^-1^K^-1^ for lysozyme and RNase-A, respectively, in the presence of any of the urea and urea-NaCl mixtures for the evaluation of Δ*G*_D_°. At a constant concentration of each of urea or urea-NaCl mixture, Δ*G*_D_° values were estimated using Equation 2 with known values of Δ*H*_m_, *T*_m_, and *C*_p_. Since the estimation using this procedure requires large extrapolation, the Δ*G*_D_° determination may be associated with large errors. Using Becktel and Schellman's procedure [33], we have estimated the maximum and minimum errors of Δ*G*_D_° at each solvent conditions. We have three separate *T*_m_ and Δ*H*_m_ obtained from three individual measurements at each [urea] and urea-NaCl mixtures. Thus, we obtained six values of Δ*G*_D_° from three maximum and three minimum values. The average values of Δ*G*_D_° and the mean errors were estimated using all the six values of Δ*G*_D._ It was found that the mean error for the Δ*G*_D_° was 5–9% for both the proteins. These Δ*G*_D_° values are given in Table 1.

**Fig 3:**
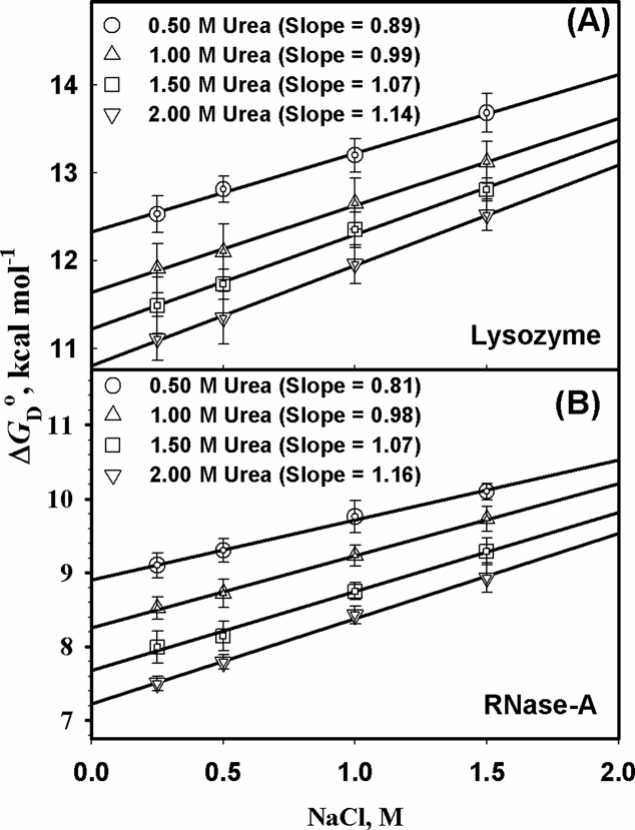
Effect of salt on protein stability in the presence of urea. Plots of ΔG_D_^0^ verses [NaCl]. Panels (A) and (B) are plots of ΔG_D_° verses [NaCl] for lysozyme and RNase-A, respectively.

**Fig 4:**
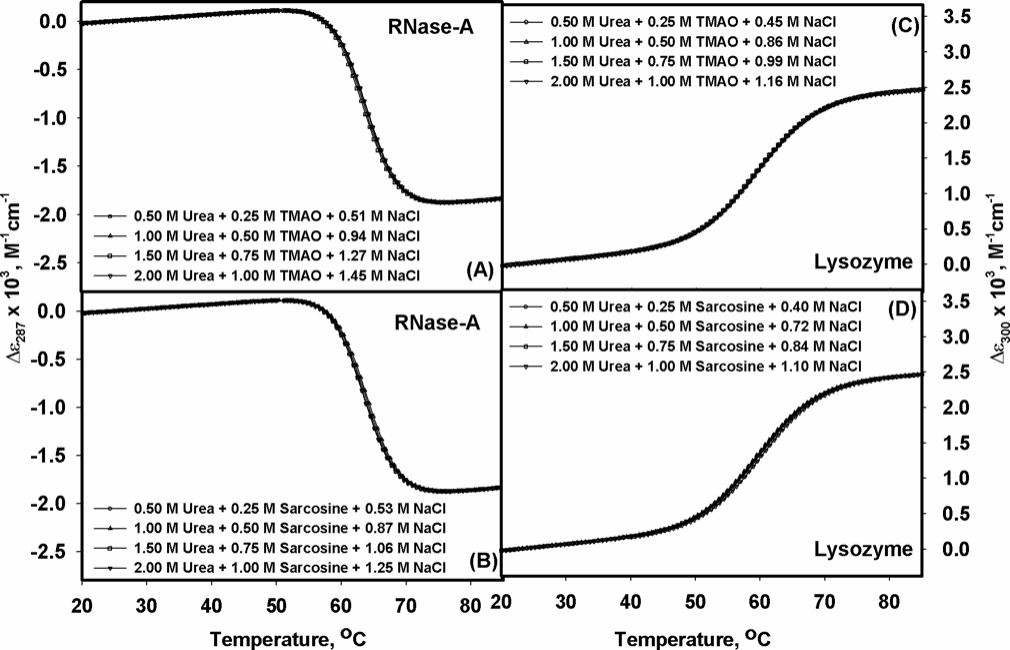
Salt induced perfect urea-methylamine counteraction. Heat-induced denaturation curves of lysozyme and RNase-A in the presence of urea-methylamine-[NaCl]^e^ mixture at pH 7.0. Concentration of each co-solvent is indicated in each panel.

Fig. 3 shows Δ*G*_D_° versus [NaCl] plots of both proteins in the presence of various fixed [urea]. Values of slope (δΔ*G*_D_°/δ [NaCl]) of both proteins in the presence of methylamine-urea mixtures are given in Table 3. This table also shows Δ*G*_D_°(Δ*G*_D_° in the presence of urea-methylamine mixture—Δ*G*_D_° in the absence of urea and methylamine). A negative value for ΔΔ*G*_D_° means that a methylamine fails to provide a perfect counteraction at 2:1 (urea: methylamine) ratio. Thus, the molar concentration of NaCl required by methylamine (in urea-methylamine mixture) to perfectly compensate the effect of urea is given as:

$${\left[\text{NaCl}\right]}^{\text{e}}=\frac{\Delta G_{\text{D}}^{0\left(\text{R}\right)}}{\Delta G_{\text{D}}^{0}/\text{ δ}\left[\text{NaCl}\right]}$$ (4)

where [NaCl]^e^ is the estimated amount of [NaCl] required for perfect counteraction at a particular urea-methylamine mixture; Δ*G*_D_^o(R)^, (Δ*G*_D_° of a protein in the absence of co-solvents—Δ*G*_D_° of a protein obtained at each urea-methylamine mixture) is the amount of Δ*G*_D_° required for a perfect counteraction, and δΔ*G*_D_°/δ[NaCl] is the slope of the Δ*G*_D_° versus [NaCl] at a given [urea]. For example, in case of lysozyme at 2.0 M urea: 1.0 M sarcosine, there is a lag of 1.65 kcal mol^-1^ (see Table 1), which is removed if this urea-sarcosine mixture contains 1.45 M NaCl (1.65 / slope of the line for 2.0 M urea (see Table 3) that is 1.14). The required salt concentrations at each [urea] estimated in this manner are given in Table 3. Therefore, ideally a perfect counteraction at any urea-methylamine ratio on protein stability is expected to be observed in the presence of respective [NaCl]^e^. We again experimentally tested if the predicted [NaCl]^e^ is really true for a perfect counteraction by measuring thermal transition curves of lysozyme and RNase-A at each urea-methylamine mixture in the presence of the respective [NaCl]^e^ and the results are given in Table 4. These denaturation transition curves are shown in Fig. 4. The thermodynamic parameters, *H*_m_ and *T*_m_ evaluated by analyzing these curves using the procedures described in the earlier section are also given in Table 4. It is evident from Fig. 4 and Table 4 that all the transition curves, and hence the thermodynamic parameters for both the proteins are, within experimental errors, identical to that in the absence of any co-solvents. Thus, urea-methylamine system comes to perfect counteraction of the protein stability in the presence of salt.

**Table 3:** Predicted NaCl concentration required for the perfect counteraction.

Concentration, (M)	^a^ΔΔ*G*_D_° (kcal mol^-1^)	δΔ*G*_D_°/δ[NaCl] (kcal mol^-1^/M)	[NaCl]^b^ (M)
	**Lysozyme** **[Urea + TMAO]**		
0.50 + 0.25	-0.26	0.89	0.29
1.00 + 0.50	-0.48	0.99	0.48
1.50 + 0.75	-1.19	1.07	1.11
2.00 + 1.00	-1.58	1.14	1.39
	**[Urea + Sarcosine]**		
0.50 + 0.25	-0.38	0.89	0.43
1.00 + 0.50	-0.62	0.99	0.63
1.50 + 0.75	-1.29	1.07	1.21
2.00 + 1.00	-1.65	1.14	1.45
	**RNase A** **[Urea + TMAO]**		
0.50 + 0.25	-0.41	0.81	0.51
1.00 + 0.50	-0.99	0.98	1.01
1.50 + 0.75	-1.44	1.07	1.35
2.00 + 1.00	-1.68	1.16	1.45
	**[Urea + Sarcosine]**		
0.50 + 0.25	-0.43	0.81	0.53
1.00 + 0.50	-0.91	0.98	0.93
1.50 + 0.75	-1.2	1.07	1.12
2.00 + 1.00	-1.45	1.16	1.25

^a^Δ*G*_D_° that should be raised for perfect counteraction.

^b^Estimated amount of [NaCl] required for perfect counteraction.

**Table 4:** Thermodynamic parameters associated with thermal unfolding of lysozyme and RNase-A in the presence of urea-methylamine-NaCl^b^ mixture at pH 7.0.

Concentration M	*T*_m°_C	Δ*H*_m_ kcal mol^-1^
	**Lysozyme** **Urea + TMAO + NaCl**	
0.50 + 0.25 + 0.29	86.0 ± 0.3	132 ± 4
1.00 + 0.50 + 0.48	86.3 ± 0.2	133 ± 2
1.50 + 0.75 + 1.11	86.2 ± 0.1	132 ± 3
2.00 + 1.00 + 1.39	86.2 ± 0.2	131 ± 3
	**Urea + Sarcosine + NaCl**	
0.50 + 0.25 + 0.43	86.5 ± 0.2	132 ± 3
1.00 + 0.50 + 0.63	86.5 ± 0.3	131 ± 2
1.50 + 0.75 + 1.21	86.4 ± 0.1	133 ± 4
2.00 + 1.00 + 1.45	86.3 ± 0.2	133 ± 2
	**RNase-A** **Urea + TMAO + NaCl**	
0.50 + 0.25 + 0.51	63.7 ± 0.2	113 ± 3
1.00 + 0.50 + 1.01	63.5 ± 0.1	114 ± 2
1.50 + 0.75 + 1.35	63.5 ± 0.3	114 ± 4
2.00 + 1.00 + 1.45	63.6 ± 0.2	115 ± 4
	**Urea + Sarcosine + NaCl**	
0.50 + 0.25 + 0.53	63.5 ± 0.1	114 ± 2
1.00 + 0.50 + 0.93	63.7 ± 0.3	113 ± 3
1.50 + 0.75 + 1.12	63.6 ± 0.2	113 ± 3
2.00 + 1.00 + 1.25	63.4 ± 0.3	113 ± 4

Estimated amount of [NaCl] required for perfect counteraction.

We also investigated if the observed thermodynamic effects for the possible involvement of NaCl are indeed true by measuring the functional activity of lysozyme and RNase-A at each urea-methylamine or urea-methylamine-NaCl mixture as given in Table 4. The functional activity of lysozyme mediated lysis of *M*. *luteus* cell wall and RNase-A mediated hydrolysis of cytidine mono phosphate (C > p) were carried out in the absence and presence of urea-methylamine or urea-methylamine-[NaCl]^e^ mixtures. The observed kinetic parameters (*K*_m_ and *k*_cat_) of lysozyme and RNase-A activity are presented in Tables 5 and 6, respectively. Values of kinetic parameters in Table 5 and 6 represent the mean of three independent measurements. It is seen in this table that there is a weak counteraction between urea and methylamines on the kinetic parameters (either *K*_m_ or *k*_cat_) at each ratio tested. But nearly complete counteraction of the parameters (*K*_m_ and *k*_cat_) is seen if the urea-methylamine mixture contains NaCl. S2 Table also shows the effect of different concentrations of NaCl alone (in the absence of urea and methylamine) on the *K*_m_ and *k*_cat_ of the proteins. It is seen in this table that enzyme activity (in terms of *K*_m_ and *k*_cat_) is inhibited at higher NaCl concentrations.

**Table 5:** Activity parameters of lysozyme in the presence and absence of urea, methylamines and urea-methylamine-[NaCl]^b^ mixture at pH 7.0 and 25°C.

[Co-solvent], M	[NaCl]^b^ (M)	*K*_m_ (μg ml^-1^)	*k*_cat_ (mg ml^-1^ s^-1^ M^-1^)	*k*_cat_/*K*_m,_ (X 10^3^, M^-1^ s^-1^)
0.00	-	77.8 ± 0.3	484.1 ± 0.3	6.22 ± 0.02
		**Urea**		
0.50	-	87.3 ± 0.6	458.6 ± 0.2	5.25 ± 0.04
1.00	-	93.4 ± 0.4	435.0 ± 0.1	4.71 ± 0.02
1.50	-	97.6 ± 0.5	375.6 ± 0.4	3.85 ± 0.02
2.00	-	107.7 ± 0.7	345.2 ± 0.3	3.20 ± 0.02
		**TMAO**		
0.25	-	76.9 ± 0.3	520.5 ± 0.5	6.77 ± 0.03
0.50	-	70.6 ± 0.7	533.0 ± 0.3	7.55 ± 0.08
0.75	-	66.4 ± 0.4	561.4 ± 0.7	8.45 ± 0.05
1.00	-	62.4 ± 0.5	566.5 ± 0.9	9.08 ± 0.07
		**Sarcosine**		
0.25	-	75.5 ± 0.6	557.9 ± 0.6	7.39 ± 0.06
0.50	-	68.2 ± 0.5	564.5 ± 0.2	8.28 ± 0.06
0.75	-	63.7 ± 0.9	571.6 ± 0.3	8.97 ± 0.02
1.00	-	59.8 ± 0.4	588.8 ± 0.5	9.85 ± 0.06
		**Urea + TMAO**		
0.50 + 0.25	-	82.3 ± 0.7	474.4 ± 0.2	5.76 ± 0.05
1.00 + 0.50	-	87.0 ± 0.8	443.6 ± 0.5	5.10 ± 0.05
1.50 + 0.75	-	91.6 ± 0.5	423.8 ± 0.2	4.63 ± 0.03
2.00 + 1.00	-	96.6 ± 0.3	408.4 ± 0.6	4.23 ± 0.02
		**Urea + Sarcosine**		
0.50 + 0.25	-	80.9 ± 0.3	477.8 ± 0.2	5.91 ± 0.02
1.00 + 0.50	-	84.4 ± 0.7	447.4 ± 0.4	5.30 ± 0.04
1.50 + 0.75	-	87.1 ± 0.2	431.5 ± 0.3	4.95 ± 0.01
2.00 + 1.00	-	91.4 ± 0.6	413.9 ± 0.2	4.53 ± 0.03
		**Urea + TMAO + NaCl**		
0.50 + 0.25	0.29	77.5 ± 0.4	479.7 ± 0.2	6.19 ± 0.03
1.00 + 0.50	0.48	77.7 ± 0.2	481.0 ± 0.2	6.19 ± 0.02
1.50 + 0.75	1.11	78.1 ± 0.3	484.2 ± 0.5	6.20 ± 0.03
2.00 + 1.00	1.39	78.0 ± 0.2	484.4 ± 0.4	6.21 ± 0.02
		**Urea + Sarcosine + NaCl**		
0.50 + 0.25	0.43	78.0 ± 0.3	482.8 ± 0.4	6.19 ± 0.02
1.00 + 0.50	0.63	77.8 ± 0.3	480.8 ± 0.3	6.18 ± 0.03
1.50 + 0.75	1.21	78.2 ± 0.2	486.4 ± 0.5	6.22 ± 0.02
2.00 + 1.00	1.45	77.6 ± 0.3	485.0 ± 0.4	6.25 ± 0.03

^b^Estimated amount of salt required for perfect counteraction, determined from thermodynamic measurements

**Table 6:** Activity parameters of RNase-A in the presence and absence of urea, methylamines and urea-methylamine-[NaCl]^b^ mixture at pH 7.0 and 25°C.

[Co-solvent], M	[NaCl]^b^ (M)	*K*_m,_ (mM)	*k*_cat_ (s^-1^)	*k*_cat_/*K*_m_ (X 10^3^, M^-1^ s^-1^)
0.00	-	0.99 ± 0.02	3.44 ± 0.08	3.48 ± 0.11
		**Urea**		
0.50	-	1.16 ± 0.03	3.22 ± 0.10	2.78 ± 0.11
1.00	-	1.36 ± 0.04	3.02 ± 0.06	2.22 ± 0.08
1.50	-	1.49 ± 0.02	2.75 ± 0.07	1.85 ± 0.05
2.00	-	1.69 ± 0.03	2.48 ± 0.09	1.47 ± 0.06
		**TMAO**		
0.25	-	0.90 ± 0.03	3.59 ± 0.05	3.99 ± 0.14
0.50	-	0.79 ± 0.04	3.71 ± 0.06	4.70 ± 0.25
0.75	-	0.71 ± 0.03	3.84 ± 0.06	5.41 ± 0.24
1.00	-	0.62 ± 0.05	3.96 ± 0.05	6.39 ± 0.52
		**Sarcosine**		
0.25	-	0.86 ± 0.04	3.63 ± 0.06	4.22 ± 0.21
0.50	-	0.73 ± 0.02	3.79 ± 0.05	5.19 ± 0.16
0.75	-	0.65 ± 0.03	3.90 ± 0.05	6.00 ± 0.29
1.00	-	0.56 ± 0.03	4.03 ± 0.07	7.20 ± 0.40
		**Urea + TMAO**		
0.50 + 0.25	-	1.07 ± 0.03	3.29 ± 0.10	3.08 ± 0.13
1.00 + 0.50	-	1.27 ± 0.02	3.07 ± 0.11	2.42 ± 0.09
1.50 + 0.75	-	1.39 ± 0.05	2.80 ± 0.12	2.01 ± 0.11
2.00 + 1.00	-	1.54 ± 0.04	2.57 ± 0.09	1.67 ± 0.07
		**Urea + Sarcosine**		
0.50 + 0.25	-	1.03 ± 0.04	3.31 ± 0.08	3.21 ± 0.15
1.00 + 0.50	-	1.23 ± 0.03	3.13 ± 0.09	2.54 ± 0.09
1.50 + 0.75	-	1.35 ± 0.05	2.83 ± 0.08	2.10 ± 0.10
2.00 + 1.00	-	1.49 ± 0.04	2.68 ± 0.06	1.80 ± 0.06
		**Urea + TMAO + NaCl**		
0.50 + 0.25	0.51	1.00 ± 0.03	3.46 ± 0.04	3.46 ± 0.11
1.00 + 0.50	1.01	1.00 ± 0.02	3.45 ± 0.02	3.45 ± 0.07
1.50 + 0.75	1.34	0.99 ± 0.02	3.43 ± 0.03	3.46 ± 0.08
2.00 + 1.00	1.45	0.98 ± 0.03	3.42 ± 0.05	3.49 ± 0.12
		**Urea + Sarcosine + NaCl**		
0.50 + 0.25	0.53	0.99 ± 0.02	3.45 ± 0.03	3.48 ± 0.08
1.00 + 0.50	0.92	0.98 ± 0.02	3.44 ± 0.04	3.51 ± 0.08
1.50 + 0.75	1.12	1.01 ± 0.03	3.49 ± 0.05	3.46 ± 0.11
2.00 + 1.00	1.25	1.03 ± 0.02	3.54 ± 0.06	3.44 ± 0.09

^b^Estimated amount of salt required for perfect counteraction, determined from thermodynamic measurements.

## Discussion

Several inorganic salts favor compact protein conformations because of preferential exclusion from the protein surface: this makes them good stabilizers of both the native and partially folded states [34]. Since NaCl is found abundantly in all urea-rich cells and has a good correlation with rise and fall of urea concentration as a consequence of urine concentrating mechanism of mammalian kidney cells, we thought it would be worthwhile to investigate if NaCl can alleviate methylamine’s ability (in terms of thermodynamic stability) to offset the deleterious effect of urea and bring about perfect counteraction. To examine for the possible role of NaCl, we intentionally carried out measurement of heat-induced transition curves of lysozyme and RNase-A in the presence of various urea-NaCl mixtures. In the analysis of transition curves, it was assumed that denaturation follows a two state unfolding mechanism. Based on Differential Scanning Calorimetric (DSC) measurements, the assumption is indeed true for these proteins in the absence of osmolytes. To examine if the denaturation curves follow a two state mechanism in the presence of urea-NaCl mixture, we have carried out the heat-induced denaturation of lysozyme and RNase in the presence of 0.5 M urea and 0.5 M NaCl and their mixture (0.5 M urea + 0.5 M NaCl) using two different probe, Δ (that measures tyrosine/tryptophan microenvironment) and [*θ*]_222_ (that measures the change in secondary structure contents). It was found that the two different probes gave identical values of *T*_m_ and Δ*H*_m_ and hence a two-state assumption is valid. To our surprise we observed that urea decreases *T*_m_ and Δ*G*_D_° on both proteins while addition of NaCl to a large extent reverses the attenuation in both *T*_m_ and Δ*G*_D_° caused by urea. Thus, similar to methylamines, NaCl also has the ability to protect proteins from destabilization by urea. Therefore, it seems most probable that the two osmolytes work in association to protect proteins from the denaturing effect of urea. If this argument is really true, it is then expected that the thermodynamic effect of the combination of both NaCl and methylamine should be additive and therefore, one can easily predict the required amount of salt to yield complete thermodynamic counteraction of the urea’s effect after taking into consideration of the extent of counteraction offered by methylamine osmolyte against urea destabilization. In order to examine the possibility we first of all estimated the exact amount of [NaCl] required for perfect counteraction from the slope of Δ*G*_D_° versus [NaCl] plots (see Fig. 3). We again experimentally tested if the prediction holds true by measuring thermal transition curves in the presence of specific concentrations of urea, methylamine and the predicted [NaCl]. Thermal transition curves given in Fig. 4 and thermodynamic parameters given in Table 4 indicate that the observation for the possible involvement of salt in the urea-methylamine counteraction of the protein is indeed real. Addition of the [NaCl]^e^ at each urea-methylamine mixture yields thermodynamic parameters (*T*_m_ and Δ*H*_m_) equal to that of control (in absence of any co-solute). We therefore conclude that NaCl can further stabilize lysozyme and RNase-A structure at each urea-methylamine mixture.

Since, thermodynamic parameters observed here are physical quantities it is important to validate our results based on how the enzyme function’s under the given solvent conditions. It is expected that urea must increase *K*_m_ and decrease *k*_cat_ because of decrease in the native molecules caused by shifting the denaturation equilibrium N ⇔ D state toward right while methylamine-[NaCl]^e^ mixture must have the opposite effect on the parameters. Therefore, we have intentionally measured *K*_m_ and *k*_cat_ of lysozyme and RNase-A in the presence of specific concentrations of the methylamine-[NaCl]^e^ mixtures. Measurements of *K*_m_ and *k*_cat_ of lysozyme mediated lysis of *M*. *luteus* cell wall (Table 5) and RNase-A mediated hydrolysis of cytidine 2′-3′ cyclic monophosphate (Table 6) in the presence of urea, urea-methylamine and urea-methylamine-NaCl mixtures suggest that, as expected, urea increases *K*_m_ and decreases *k*_cat_, methylamines weakly counteract the urea’s effect on *K*_m_ and *k*_cat_ and addition of the [NaCl]^e^ at each urea-methylamine mixture brings values of *K*_m_ and *k*_cat_ to near control values (in the absence of any co-solutes). Our observed effects of the role of NaCl to counter urea-induced protein destabilization and enzyme inactivation are in good agreement with various studies on cell lines. Several cell line studies support the possible involvement of NaCl in counteracting urea’s effect. For instance (i) the decrease in growth of mIMCD cells caused by 250 mM urea is completely reversed upon treatment with NaCl [35], (ii) pretreatment with hypertonic NaCl also protects another cell line, MDCK cells against high urea concentrations [36] and (iii) in a reverse experiment, urea also protects from the apoptotic effects of NaCl in renal medullary cells [35].

Another aspect of this counteraction system that needs to be explored is the mechanism behind NaCl mediated potentiation of methylamines to completely counteract the deleterious effect of urea on protein stability and activity. The innocuous effect of salt in urea-methylamine mixture on the observed overall activity of RNase-A and lysozyme clearly indicates that similar to the effect of osmolytes which are preferentially excluded from the protein domains, the NaCl (in presence of methylamines) perhaps do not have direct interactions with the protein *par se*. A support to this possibility comes from the earlier report that monovalent salts are also good protein stabilizers by their preferential exclusiveness from the protein surface [34,37,38]. Interestingly, in one study, folding and unfolding kinetics of c-terminal-NPM1 (nucleophosmin 1) in the presence of different sodium chloride concentrations ranging from 0.075 to 1 M were studied at pH 7.0 [39]. It was observed that there was 5 fold increase in the rate of refolding ((*k*_F_) s^-1^) and also the same fold decrease in the unfolding rate ((*k*_u_) s^-1^) in the presence of 1.0 M NaCl. The increase in the rate of refolding was due to the formation of pre-organized structure induced by NaCl to the denatured state of the protein. It has also been known that the methylamine’s ability to induce protein folding is due to the unfavorable interaction with the peptide backbone. Interactions with the side chains (although little) are favorable and therefore, oppose the unfavorable interaction. Since NaCl induces pre-organized structure in the denatured state of protein [39], it is quite possible that the NaCl-induced pre-organized structures might have more exposed peptide backbone and less solvent exposed side chains resulting in enhance unfavorable (osmophobic) interaction and hence preferential hydration.

In summary, we are sure that (i) methylamines alone gives weak/partial counteraction of effects of urea on proteins while for a complete counteraction, two osmotic components NaCl and methylamines mixture are required, and (ii) cellular salts may modulate the chaperonic effect of charged/zwitterionic osmolytes so as to enhance the rate of refolding. Our study therefore indicates that salt-mediated modulation of protein-osmolyte interactions and hence in regulation of protein function must be widely investigated.

## Supporting Information

S1 Table Thermodynamic parameters associated with the thermal unfolding of lysozyme and RNase-A in the presence of different NaCl concentrations at pH 7.0. (DOCX)

S2 Table Activity parameters of lysozyme and RNase-A in the presence and absence of different NaCl concentrations at pH 7.0 and 25°C. (DOCX)
