# Supplementary material for: Salt Potentiates Methylamine Counteraction System to Offset the Deleterious Effects of Urea on Protein Stability and Function
Source: PLoS One. 2015 Mar 20;10(3):e0119597. doi: 10.1371/journal.pone.0119597 (PMC4368626; doi:10.1371/journal.pone.0119597)
Supplement: S1 Table — (DOCX) [file pone.0119597.s001.docx]

| **Lysozyme** | | | | **RNase-A** | | |
| --- | --- | --- | --- | --- | --- | --- |
| **NaCl** | ***T*_m_** | **Δ*H*_m_** |  |  | ***T*_m_** | **Δ*H*_m_** |
| **(M)** | **(^o^C)** | **(kcal/mol)** |  |  | **(^o^C)** | **(kcal/mol)** |
| 0.00 | 86.0 ± 0.2 | 130 ± 3 |  |  | 63.6 ± 0.3 | 114 ± 2 |
| 0.50 | 85.9 ± 0.4 | 131 ± 4 |  |  | 64.0 ± 0.1 | 116 ± 3 |
| 1.00 | 86.7 ± 0.3 | 133 ± 3 |  |  | 64.8 ± 0.2 | 118 ± 3 |
| 1.50 | 87.2 ± 0.1 | 132 ± 2 |  |  | 65.6 ± 0.4 | 119 ± 4 |
| 2.00 | 88.1 ± 0.2 | 135 ± 4 |  |  | 66.5 ± 0.3 | 121 ± 3 |
