# Supplementary material for: Salt Potentiates Methylamine Counteraction System to Offset the Deleterious Effects of Urea on Protein Stability and Function
Source: PLoS One. 2015 Mar 20;10(3):e0119597. doi: 10.1371/journal.pone.0119597 (PMC4368626; doi:10.1371/journal.pone.0119597)
Supplement: S2 Table — (DOCX) [file pone.0119597.s002.docx]

|  | **Lysozyme** |  |  | **RNaseA** |  |
| --- | --- | --- | --- | --- | --- |
| **[NaCl]*** | ***K*_m_** | ***k*_cat_** | **[NaCl]** | ***K*_m_** | ***k*_cat_** |
| **(M)** | **μg ml^-1^** | **mg ml^-1^ s^-1^ M^-1^** | **(M)** | **(mM)** | **(s^-1^)** |
| 0.00 | 77.89 ± 0.3 | 484 ± 0.3 | 0.00 | 0.99 ± 0.02 | 3.44 ± 0.08 |
| 0.10 | 102.4 ± 1.7 | 406 ± 2.6 | 0.25 | 1.02 ± 0.05 | 3.40 ± 0.12 |
| 0.20 | 122.3 ± 2.7 | 314 ± 3.1 | 0.50 | 1.19 ± 0.07 | 2.98 ± 0.11 |
| 0.30 | 137.2 ± 3.6 | 253 ± 3.1 | 0.75 | 1.23 ± 0.03 | 2.62 ± 0.10 |
| 0.40 | ND^#^ | ND | 1.00 | 1.37 ± 0.06 | 2.37 ± 0.13 |
| 1.00 | ND | ND | 1.50 | ND | ND |
|  |  |  | 2.00 | ND | ND |
|  |  |  |  |  |  |

^*^We have taken NaCl concentrations different from that used in case of RNase-A because NaCl at high concentrations inhibits lysozyme; ^#^Not determined
